# Notes and News

**Published:** 1901-03-02

**Authors:** 


### Notes and News.

HOSPITAL MEETINGS.

London Hospital.—Annual meeting, Wednesday, February 6th, 3.30.
City of London Hospital for Diseases of the Chest.—Annual meeting, Gresham College, Thursday, March 7th, 4.30.
New Hospital for Women.—Annual meeting, Friday, March 8th, 3 p.m.

Queen Alexandra has intimated that her Presidency of Cheyne Hospital for Children, Cheyne Walk, Chelsea, will still continue.

Sir Samuel Montagu, Bart., has accepted the hon. treasurership of the Italian Hospital, and Sir Dyce Duckworth, M.D., LL.D., has joined the consulting staff.

The lecturers and honorary staff of the Dental Hospital of London have contributed a special donation of £2,000 towards the building of the new hospital in Leicester Square, which is now nearing completion.

The Royal Alfred Aged Merchant Seamen's Institution has just received a donation of £1,000 from its president, Mr. R. S. Donkin, and his wife, enabling the committee to nominate fifteen of the aged applicants to receive the out-pension of this national institution for worn-out British merchant seamen in addition to the twenty elected by the votes of the subscribers and donors

It has often struck us that the obligation newspaper editors and proprietors are under to the distributors of newspapers might be best met by each newspaper establishing a certain number of pensions for men and women in connection with the Newsvendors' Benevolent and Provident Institution. In fulfilment of this idea we arranged last year to give two such pensions, to be known as "The Hospital" Pensions, and the committee of the Newsvendors' Benevolent Institution have informed us that the recipients for the present year are Charles Keats, who after forty years' service in the printing trade is now nearly blind and so unable to support himself; and Mrs. Easton (the widow of a late employé in the office of the *Morning Post*), who is a confirmed invalid. We hope that before many years have passed the list of donors of pensions distributed through the Newsvendors' Benevolent Institution will contain the name of every newspaper published in the United Kingdom for at least one such pension.

The English visitors at Mentone are sending 40,000 lemons for the use of soldiers in hospital in South Africa.

By the will of the late Miss Annie Downing, of Enfield, the Brompton Cancer Hospital, the London Temperance Hospital, and the Mildmay Nursing Home, are to participate in the residue of her estate, amounting to a sum of £10,000.

The infected areas of the plague continue to spread, though in most cases, like at the Cape, watchful care prevents the visitations from assuming the terrible proportions which the epidemic has always assumed in India. The news from Bombay is still exceedingly grave, hundreds of fatal cases being reported daily. In Singapore three cases are recorded.

Mr. Richard Copley Christie, of Ribsden Windlesham, Surrey, who died on January 9th, has left £1,000 to the following institutions:—The Manchester Royal Infirmary, the Manchester Southern Hospital for Women and Children, the Clinical Hospital for Women and Children at Chesham, and the Cancer Pavilion Aid Home, in Hospital Gardens, Manchester.

At the annual Central Poor Law Conference this week a resolution was submitted to the effect that it was desirable that the care of feeble-minded and epileptic children should devolve upon the county councils. To this an amendment that these classes should be cared for by boards of guardians in special institutions, and not in workhouses, was moved, and carried by a large majority.

The foundation-stone of a new lunatic asylum for the county of Middlesex was laid on Tuesday by the chairman of the Asylum Committee. The site of the building is 440 acres in extent, and was purchased at a cost of £45,000. It is situated just outside St. Albans. The plans are by Mr. Rowland Plumbe, who has designed the buildings in the pavilion style to accommodate 1,150 patients, 100 of whom will be in a separate building, which is to be used only by paying patients. A hospital to accommodate 234 persons is included. The estimated cost of the buildings alone is £358,100, or £311 per patient.

It has come to the knowledge of a few friends of the late Dr. A. H. Jacob, Irish Editor of the *Medical Press and Circular*, that owing to a long continued period of ill-health, and other causes, he has died leaving his wife and daughters insufficiently provided for. For over thirty years Dr. Jacob was one of the most earnest workers on the Council of the Irish Medical Association, and for the same period was connected with the Royal College of Surgeons, and at the time of his death was senior member of the Council. For many years he proved a fearless and able advocate of the rights of the Poor Law Medical Practitioners of Ireland in the effort to obtain for them an adequate salary, security of tenure, and a pension in old age. A committee has been formed, with power to add to their number, for the purpose of collecting subscriptions for the "Jacob Memorial Fund." Through this means the profession and the public have now an opportunity of helping materially his widow and daughters in a way in which he himself was not permitted to do, owing to ill-health and an unselfish life-long service in the interests of the profession. Cheques and Postal Orders may be made payable to hon. treasurers, one of whom is The Registrar, Royal College of Surgeons, Dublin.

